# Bacteriocins in Veterinary Medicine: From Antibiotic Limitations to Targeted Solutions

**DOI:** 10.3390/ijms27135812

**Published:** 2026-06-27

**Authors:** Marta Książczyk, Katarzyna Dębowska, Karolina Bierowiec

**Affiliations:** 1 Department of Epizootiology and Clinic of Birds and Exotic Animals, Faculty of Veterinary Medicine, Wrocław University of Environmental and Life Sciences, Grunwaldzki Sq. 45, 50-366 Wrocław, Poland; marta.ksiazczyk@uwr.edu.pl (M.K.); karolina.bierowiec@upwr.edu.pl (K.B.); 2 Department of Microbiology, Faculty of Biological Sciences, University of Wrocław, Przybyszewskiego 63, 51-148 Wrocław, Poland; 3 EZA Student Science Club, Department of Epizootiology and Clinic of Birds and Exotic Animals, Faculty of Veterinary Medicine, Wrocław University of Environmental and Life Sciences, Grunwaldzki Sq. 45, 50-366 Wrocław, Poland

**Keywords:** bacteriocins, veterinary medicine, antimicrobial resistance, biofilm, MRSA, MRSP, mastitis

## Abstract

Antimicrobial resistance (AMR) has emerged as one of the foremost global threats to public health, with the veterinary sector, responsible for nearly three-quarters of global antimicrobial consumption, representing an underappreciated epicenter of this crisis. Despite the extensive literature on bacteriocins as antibiotic alternatives, most reviews focus on human medicine or food preservation, leaving a conspicuous gap in evidence specific to veterinary medicine. The present review addresses this gap by examining the molecular basis of bacteriocin activity (lipid II, bacterial RNA polymerase, cytoplasmic membrane), strategies for clinical deployment (topical therapy, antibiotic combinations, disruption of biofilm tolerance), and preclinical evidence relevant to bovine mastitis, canine pyoderma and otitis externa, and infections caused by multidrug-resistant pathogens (MRSA (Methicillin-Resistant *Staphylococcus aureus*), MRSP (Methicillin-Resistant *Staphylococcus pseudintermedius*), colistin-resistant *P. aeruginosa*, XDR (extensively drug-resistant) *Acinetobacter baumannii*). Translational barriers—pharmacokinetic, regulatory, and evidentiary—are critically appraised, alongside emerging directions including precision nanocarriers, biofilm-targeted therapies, and the animal microbiota as a reservoir of novel molecules. Bacteriocins represent a promising yet underexploited antibacterial class in response to the escalating AMR crisis in the animal sector.

## 1. Introduction

Antimicrobial resistance (AMR) has been listed by the World Health Organization (WHO) among the ten leading global threats to public health. According to the Global Burden of Disease (GBD) study, AMR was the direct cause of 1.27 million deaths and associated with a further 4.95 million deaths worldwide in 2019 [1], and the most recent projections estimate that the annual mortality directly attributable to AMR will rise to 1.91 million by 2050 [2]. In the European Union and the European Economic Area, antimicrobial-resistant infections account for over 35,000 deaths per year and generate societal costs on the order of EUR 1.5 billion [3,4]. Although AMR is most often framed in public discourse as a problem of human medicine, its origins and dynamics are closely linked to the animal sector.

Among the proposed alternatives to conventional antibiotics, bacteriocins—ribosomally synthesized antibacterial peptides produced by bacteria—have emerged as one of the most extensively investigated classes, particularly in the veterinary setting; this review surveys their molecular basis, application strategies, and translational hurdles. Recent structural and biophysical advancements have greatly enhanced our understanding of these peptides, revealing the complex protein–protein interactions and transport mechanisms they use to navigate and subvert the bacterial cell envelope [5].

## 2. Therapeutic Bottlenecks in Veterinary Medicine

The scale of antimicrobial use in livestock production is central to this picture: approximately 73% of all antibiotics consumed globally are administered to food-producing animals rather than to humans [6,7], and Livestock Biomass Conversion projections indicate that global veterinary consumption will rise by nearly 30% by 2040 under a business-as-usual scenario [8]. More importantly, the five antimicrobial classes most widely used in veterinary medicine—tetracyclines, β-lactams, macrolides, sulfonamides, and fluoroquinolones—overlap with those classified by the WHO as Highest Priority Critically Important Antimicrobials in human medicine [9,10]. The consequences of this overlap are well documented: the use of ceftiofur in poultry hatcheries has selected for β-lactamases in *Salmonella enterica* serovar Heidelberg that confer cross-resistance to ceftriaxone—a first-line agent for invasive salmonellosis in humans [11]. This example illustrates the fundamental premise of the One Health framework: the therapeutic arsenals of veterinary and human medicine are eroded jointly and inseparably [12].

Despite this shared pool of active substances, veterinary medicine has access to a markedly narrower portfolio of registered antimicrobials than human medicine. The therapeutic bottlenecks discussed in this section arise from overlapping structural, biological, and regulatory constraints, and it is precisely these constraints that drive the search for alternatives, including bacteriocins.

### 2.1. Limited Therapeutic Options

A fundamental constraint on therapeutic options in veterinary medicine is the species-specific nature of drug authorization: antimicrobials are licensed for defined indications and target species, and for many pathogen–species combinations, no effective registered therapy exists. Veterinary clinicians therefore frequently resort to off-label prescribing, regulated within the European Union under the so-called cascade (Regulation (EU) 2019/6), which carries the inherent risks of suboptimal dosing and therapeutic failure [10,13].

The World Organization for Animal Health (WOAH) classifies veterinary antimicrobials according to their clinical importance, defining a category of Veterinary Critically Important Antimicrobials (VCIAs). Within this group, four classes—fluoroquinolones, third- and fourth-generation cephalosporins, colistin, and fosfomycin—are additionally designated as Highest Priority Critically Important Antimicrobials (HPCIAs), reflecting their pivotal role in human medicine. Selected clinical indications for which no equivalent therapeutic alternatives are currently available are summarized in Table 1 [13].

This situation is particularly concerning given the rising prevalence of bacterial resistance and the expansion of the so-called animal resistome—the pool of resistance genes harbored by both pathogenic and commensal bacteria. There is consistent evidence that the use of antimicrobials in veterinary medicine, including at subtherapeutic doses, promotes the selection and dissemination of resistance genes, with their diversity and abundance correlating with the intensity of antimicrobial use [12]. In addition, the low oral bioavailability of many veterinary antimicrobials in livestock (1–20%) results in a substantial fraction of the administered dose being excreted unchanged in a biologically active form (tetracyclines: 25–80%; macrolides: 40–100%; β-lactams: up to 90%), thereby intensifying environmental selection pressure [14]. In countries with weaker regulatory frameworks, this is further compounded by over-the-counter availability of antimicrobials and the circulation of substandard products, leading to prolonged exposure of bacterial populations to subtherapeutic concentrations—conditions that are particularly conducive to resistance selection [15]. Veterinary clinicians are therefore increasingly forced to choose between treatments of uncertain efficacy and no treatment at all—a clinical reality that directly motivates the search for new classes of antibacterial agents, including bacteriocins.

### 2.2. Biofilm-Associated Infections

Biofilms represent the predominant mode of bacterial existence in natural environments, with an estimated 80% of microbial biomass in the biosphere residing within multicellular communities embedded in an extracellular matrix rather than as free-floating planktonic cells [16]. Under livestock husbandry conditions, biofilms readily develop on the surfaces of milking equipment, feeding systems, floors, and watering installations, as well as on the skin, glands, and mucosal surfaces of the animals themselves [17].

From a pathophysiological standpoint, a biofilm is a multicellular structure encased in a self-produced matrix of extracellular polymeric substances (EPSs), composed of polysaccharides, proteins, lipids, and extracellular DNA (eDNA). The EPS matrix may account for over 90% of the mass of a mature biofilm and constitutes both a physical and biochemical barrier to antibiotic penetration [18]. Within biofilms, bacterial cells exhibit 10- to 1000-fold reduced susceptibility to antimicrobials compared with their planktonic counterparts. This reduced susceptibility arises from a combination of factors: restricted diffusion of antimicrobial agents through the EPS, oxygen and nutrient gradients, lowered metabolic activity of cells in deeper layers, and the presence of persistent cell subpopulations. The phenomenon is best described as tolerance rather than classical resistance, as it stems from phenotypic adaptation rather than genetic changes [19,20].

In veterinary medicine, biofilms play a central role in three clinically dominant infections. Bovine mastitis—the most economically burdensome disease in the dairy industry—has a substantial biofilm component: approximately 57% of *Staphylococcus aureus* and 54% of *Streptococcus uberis* isolates obtained from clinical and subclinical mastitis cases display biofilm-forming capacity within the mammary gland, directly correlating with chronicity of infection and failure of intramammary therapy [21]. Canine otitis externa, a chronic inflammation of the external ear canal, represents another archetypal example: between 40% and 95% of *Pseudomonas aeruginosa* isolates from chronic cases form biofilms, and these isolates increasingly belong to so-called high-risk sequence types (ST111, ST244) identical to those reported in human nosocomial infections [22]. The third domain encompasses implant- and surgical wound-associated infections in small animal surgery, where biofilms of *Staphylococcus pseudintermedius* on synthetic surfaces are the principal cause of persistent implant-related infections [23].

In contrast to conventional antibiotics, bacteriocins such as nisin retain activity against bacterial cells embedded within biofilms. Nisin binds to lipid II, the precursor of peptidoglycan biosynthesis, and this interaction simultaneously blocks cell wall assembly and drives the formation of pores within the cytoplasmic membrane, leading to rapid bacterial cell death [24]. Nisin and its genetically engineered derivatives reduce the viability of preformed biofilms of *S. aureus*, *S. pseudintermedius*, and MRSA recovered from cases of bovine mastitis and canine otitis externa, while combinations such as nisin/ceftiofur and nisin/vancomycin display synergistic activity capable of overcoming biofilm tolerance at concentrations unattainable with the antibiotics alone [25,26]. A second clinically documented example is lacticin 3147, a two-component lantibiotic produced by *Lactococcus lactis* DPC3147 whose mechanism relies on the cooperative action of two peptides (LtnA1 and LtnA2): the former binds lipid II, while the latter inserts into the cytoplasmic membrane and assembles ion-conducting channels at nanomolar concentrations [27]. In an in vivo study in dairy cows, lacticin 3147 was administered during the dry period as a component of an intramammary teat seal preparation; following experimental challenge with *Streptococcus dysgalactiae*, the incidence of mastitis was 6% in the treated group compared with 61% in the untreated controls [28].

### 2.3. Regulatory and Stewardship Pressure

The growing recognition of risks associated with the overuse of antimicrobials in animal production has driven a profound transformation of the regulatory landscape over the past two decades. A pivotal moment was the European Union’s 2006 ban on the use of antibiotics as growth promoters, the rationale of which was subsequently adopted at the global level through the Global Action Plan on Antimicrobial Resistance, endorsed by the 68th World Health Assembly in May 2015 (Resolution WHA68.7) [29], and through the One Health strategy of the Tripartite Organizations (FAO–WOAH–WHO), expanded in 2022 to a Quadripartite framework following the inclusion of UNEP [30]. In the United States, an analogous mechanism was implemented by the FDA (Food and Drug Administration) through Guidance for Industry Document Nos. 209 and 213 (2012–2017), which removed medically important antimicrobials from over-the-counter use as feed additives and placed their administration under veterinary oversight under the Veterinary Feed Directive [31].

The most stringent regulatory framework currently in force is that of the European Union. Regulations (EU) 2019/6 and (EU) 2019/4—applicable since 28 January 2022—prohibit the prophylactic use of antibiotics in groups of animals, restrict metaphylaxis to clinically justified cases, and authorize the Commission to reserve specific antimicrobial classes exclusively for human medicine (Article 37(5)) [32]. Commission Implementing Regulation (EU) 2022/1255, in force since 9 February 2023, designates 37 substances and substance groups (18 antibiotics, 18 antivirals, and one antiprotozoal) as reserved for human use and prohibited in veterinary medicinal products [33].

The most recent and consequential illustration of how antimicrobial resistance evolves and disseminates is provided by the case of colistin [34,35,36]. After several decades of marginal clinical relevance—withdrawn from human use in the 1970s due to its nephro- and neurotoxicity—colistin re-emerged in the twenty-first century as a last-resort antibiotic against infections caused by multidrug-resistant (MDR), extensively drug-resistant (XDR), and pan-drug-resistant (PDR) Gram-negative bacteria, including *P. aeruginosa*, *Klebsiella pneumoniae* and *Acinetobacter baumannii* [37]. In parallel, and independently of its trajectory in human medicine, colistin had been widely used in animal husbandry—primarily in pigs and poultry for the treatment of enteric infections caused by *E. coli*, as well as as a growth promoter [37]. This dual life of colistin—as an antibiotic of last resort in the clinic and a routine agent on the farm—proved unsustainable.

The first plasmid-borne mechanism of horizontal transfer of resistance to polymyxins was identified in 2016, in colistin-resistant *E. coli* strains isolated from Chinese pig farms [38,39]. The *mcr-1* gene encodes the MCR-1 enzyme, which modifies the surface of Gram-negative bacteria by attaching phosphoethanolamine groups to lipid A within the outer-membrane lipopolysaccharide. This subtle structural modification is sufficient to reduce the negative charge of the bacterial surface and to abolish the binding of colistin to its target [40,41]. Crucially, plasmid-borne *mcr-1* facilitates rapid interspecies dissemination, and to date, ten variants (*mcr-1* to *mcr-10*) have been identified in strains of *E. coli*, *K. pneumoniae*, *Enterobacter* spp., and *Salmonella* spp. [42]. As a consequence, the use of colistin has been severely restricted in the EU, and it was banned as a feed additive in China in 2017 and reclassified as a second-line veterinary agent in Japan in 2018 [39,42].

Both fosfomycin and polymyxins are classified by WOAH as Highest Priority Critically Important Antimicrobials (HPCIAs), reflecting their critical importance for both human and veterinary medicine [13]. The Farm-to-Fork strategy further envisages a 50% reduction in the sales of veterinary antimicrobials in the EU by 2030 [43], representing an unprecedented level of pressure on the animal production sector.

These regulatory changes have tangible clinical consequences. Since 2022, the use of HPCIAs in the EU is conditional on microbiological confirmation, delaying treatment initiation by the time required to obtain antimicrobial susceptibility results, and total sales of veterinary antimicrobials in the EU declined by more than 25% between 2018 and 2022 [44].

## 3. Why Bacteriocins Fit These Challenges

### 3.1. Mechanistic Basis for Activity Against Resistant Pathogens

What sets bacteriocins apart from conventional antibiotics begins at the very level of their biosynthesis. Whereas the latter are secondary metabolites assembled by enzymatic complexes such as non-ribosomal peptide synthetases (NRPSs) or polyketide synthases (PKSs), bacteriocins arise from the ribosomal translation of a precursor peptide that subsequently undergoes enzymatic post-translational modification; on this basis, they are classified as members of the RiPP superfamily (ribosomally synthesized and post-translationally modified peptides) [45,46]. In the case of lantibiotics, these modifications are catalyzed by LanB/LanC enzymes (class I) or LanM (class II) and yield the characteristic thioether residues lanthionine and methyllanthionine, together with the unsaturated amino acids dehydroalanine (Dha) and dehydrobutyrine (Dhb) [46]. The presence of multiple post-translational modifications, combined with the gene-encoded nature of the precursor peptide, renders bacteriocins particularly amenable to rational molecular engineering, in marked contrast to non-ribosomally synthesized peptide antibiotics such as polymyxins.

The best-characterized molecular mechanism is the dual mode of action of nisin against Gram-positive bacteria. Rings A and B of nisin engage the pyrophosphate moiety of lipid II—the membrane-anchored precursor of peptidoglycan biosynthesis—forming a so-called *pyrophosphate cage* stabilized by five hydrogen bonds [47,48]. This interaction simultaneously inhibits transglycosylation, a key step of cell wall biosynthesis, and serves as a docking platform for the assembly of a pore complex with a defined stoichiometry of eight nisin molecules to four lipid II molecules, leading to depolarization of the cytoplasmic membrane and rapid bacterial cell death [49,50]. Nisin binds lipid II with high affinity and forms membrane pores at nanomolar concentrations [51]. Critically, the pyrophosphate moiety of lipid II represents a conserved, non-modifiable pharmacophore: bacteria cannot alter it without compromising viability, which renders nisin active even against multidrug-resistant strains [52].

A markedly different mechanism is observed for bacteriocins acting on intracellular targets. Microcin J25, a 21-residue lasso peptide produced by *E. coli*, gains entry into susceptible cells in a two-step manner: first via the outer-membrane receptor FhuA, and then through the inner-membrane transporter SbmA. Once inside the cytoplasm, it binds within the *secondary channel* of bacterial RNA polymerase, sterically obstructing the entry of NTP substrates and preventing folding of the trigger loop, an essential step for catalysis [53,54]. Colicins, by contrast, are multi-domain bacteriocins (40–80 kDa) which, following translocation into the cytoplasm, act as DNA endonucleases, RNases, or membrane pore-forming peptides, depending on the nature of their catalytic domain [45].

This diversity of molecular targets—ranging from cell wall precursors (lipid II), through bacterial RNA polymerase and nucleic acids, to the cytoplasmic membrane itself—combined with the fact that none of these mechanisms overlap with those of conventional antibiotic classes, substantially limits the risk of cross-resistance. Importantly, bacteriocins retain activity against bacterial cells residing within biofilms, where, as discussed in a further section, conventional antibiotics show a 10- to 1000-fold reduction in efficacy. This persistence stems from several molecular features. First, the small size and amphipathic character of many bacteriocins facilitate their diffusion through the EPS matrix: under continuous-flow microscopy, nisin was shown to penetrate to the base of *S. aureus* biofilms within approximately 20 min of exposure, although its bactericidal efficacy against sessile cells remained lower than against their planktonic counterparts [25]. Second, the molecular targets of bacteriocins (lipid II, RNA polymerase) remain present and functional even in metabolically dormant persister cells, in contrast to antibiotics that require active cellular growth, such as β-lactams or fluoroquinolones [26].

### 3.2. Veterinary Application Strategies

The most clinically documented veterinary application of nisin is intramammary therapy of bovine mastitis. In a randomized trial involving 92 lactating dairy cows, intramammary infusion of nisin Z (2.5 × 10^6^ IU) achieved a clinical cure rate comparable to gentamicin (90.2% vs. 91.1%); none of the recovered *S. aureus* isolates exhibited resistance to nisin, in contrast to 82.5% penicillin resistance and 35.3% gentamicin resistance among the same isolates [55]. Residual nisin concentrations in milk became undetectable within 36 h of infusion, translating into a virtually zero withdrawal period. Nisin is rapidly degraded by mammalian gastrointestinal proteases, and its LD_50_ of 6950 mg/kg is comparable to that of sodium chloride [56]. These properties underlie its Generally Recognized As Safe (GRAS) status granted by the WHO in 1969 and the FDA in 1988, and its licensing as a food preservative in over 50 countries [26,56].

Analogous topical strategies are being developed for canine superficial pyoderma caused by methicillin-resistant *S. pseudintermedius* (MRSP). NZ2114, a plectasin-derived peptide originating from the fungal defensin of *Pseudoplectania nigrella*, exhibits potent activity against clinical *S. pseudintermedius* isolates, with a minimum inhibitory concentration (MIC) of 0.23 µM—superior to that of mupirocin (MIC = 0.25–0.5 µM) and lincomycin (MIC = 4.34–69.41 µM) [57]. In a murine superficial pyoderma model, a transdermal NZ2114 formulation incorporating *N*-methylpyrrolidone and propylene glycol as permeation enhancers effectively reduced cutaneous bacterial colonization, providing an alternative to systemic fluoroquinolones or cephalosporins classified as Highest Priority Critically Important Antimicrobials (HPCIAs) [58].

Against Gram-positive pathogens resistant to conventional antibiotics, nisin and its bioengineered derivatives retain antimicrobial activity. Nisin A is active against MRSA and vancomycin-resistant enterococci (VRE) [56]. In the context of bovine mastitis, the combination of nisin and vancomycin exhibited synergistic activity against vancomycin-intermediate *S. aureus* (VISA), reducing cell viability within preformed biofilms [59]. Bioengineered nisin derivatives combined with chloramphenicol demonstrate enhanced efficacy against biofilms of *S. aureus* SA113 (nisin V/M21V + chloramphenicol) and *S. pseudintermedius* DSM21284 (nisin I4V + chloramphenicol), strains of clinical relevance in mastitis and canine pyoderma, respectively [26]. The most advanced example is the three-component formulation (MP1-NisA-Aur) comprising nisin A, micrococcin P1 and AuresinePlus (a *Staphylococcus*-specific peptidoglycan hydrolase), which eradicated MRSA infections derived from bovine mastitis isolates in murine models [60].

Against Gram-negative pathogens, central in veterinary medicine in light of the regulatory restrictions on colistin and fluoroquinolones outlined in the previous section, nisin alone displays limited activity, as its molecular size precludes penetration of the lipopolysaccharide (LPS)-containing outer membrane [56]. This limitation can be overcome through divalent cation chelators (EDTA, Tween 80), which destabilize the LPS layer and expose the cytoplasmic membrane phospholipids. Of greater clinical relevance, however, are synergies with polymyxins. The combination of nisin A with colistin exhibited synergistic activity (FICI ≤ 0.5) against *E. coli* O157:H7, *P. aeruginosa*, *Yersinia enterocolitica* and *Salmonella choleraesuis*, lowering effective colistin concentrations from 0.12 to 0.01 µg/mL [61]. Critically, nisin at 1.5–2 mg/mL abolished colistin cytotoxicity in Vero renal epithelial cells, directly addressing the nephrotoxicity that constitutes the principal limitation of colistin therapy [61]. Synergistic nisin–colistin combinations have likewise been documented against clinical isolates of colistin-resistant *P. aeruginosa* and extensively drug-resistant (XDR) *A. baumannii*, both designated as critical-priority pathogens by the WHO [62], and nisin–polymyxin combinations have been validated against *P. aeruginosa* biofilms, including with bioengineered nisin variants [63]. The mechanistic basis of these synergies is sequential: polymyxins destabilize the outer membrane, allowing nisin to access the cytoplasmic membrane and lipid II [56]. This strategy enables clinically meaningful dose reduction of polymyxins and a corresponding decrease in the selective pressure driving the dissemination of *mcr* genes in the veterinary sector. Furthermore, minimizing colistin exposure is vital for host health; while 16S rRNA sequencing confirms that conventional doses of colistin cause a drastic collapse in the diversity of the gut microbiota, targeted bacteriocins like nisin Z and select microcins preserve overall microbial richness while keeping lactic acid bacteria populations stable [64]. This ecological harmony underscores the therapeutic value of the native microbiome, which recent shotgun metagenomic profiling has revealed to be a vast, untapped natural reservoir of biosynthetic gene clusters (BGCs) encoding novel antimicrobial peptides, particularly within the highly enriched environment of the avian cecum [65].

### 3.3. Engineering Bacteriocins for Clinical Translation

Owing to their gene-encoded nature, lantibiotics constitute ideal targets for rational molecular engineering aimed at optimizing their properties for clinical applications [66]. Bioengineering efforts have largely focused on the hinge region to optimize peptide stability and broaden the antimicrobial spectrum against pathogens [67]. Site-directed mutagenesis within this region of nisin A (such as the K22T, N20P, or M21V variants) has successfully generated derivatives exhibiting significantly enhanced activity against specific Gram-positive pathogens [67]. Furthermore, modifications of the serine residue at position 29 (generating the S29G or S29A variants) not only affected the peptide’s physicochemical properties but also broadened the activity spectrum to include selected Gram-negative bacteria, while intensifying efficacy against pathogenic strains resistant to conventional antibiotics [68].

The transition from laboratory scale to clinical-grade veterinary production nevertheless remains a major challenge, as conventional production in native producer strains is often insufficiently efficient, rendering large-scale manufacturing both technically demanding and costly [69,70]. Given the price sensitivity of the veterinary pharmaceutical market, heterologous expression systems are being intensively developed [66]. Expression of the plectasin-derived peptide NZ2114 in *Pichia pastoris* has been shown to achieve high fermentation yields while preserving potent bactericidal and synergistic activity against *S. aureus* [57]. Such platforms provide the foundation for scalable and economically viable production of alternative veterinary therapeutics.

Even the most potent bioengineered bacteriocin variants encounter significant in vivo barriers, particularly upon systemic administration: rapid proteolytic degradation, low stability, and unfavorable pharmacokinetics [69,71]. Optimized delivery systems represent a direct response to these limitations in veterinary medicine, particularly in dermatological applications [70]. A compelling example of a nanotechnology-based solution is the incorporation of nisin into electrospun nanofibers composed of poly(ethylene oxide) (PEO) and poly(D,L-lactide) (PDLLA) [71]. In a murine excisional skin infection model, these matrices provided sustained release of the active peptide and significantly reduced *S. aureus* burden in wounds compared with control groups [69]. Importantly, the dressing did not impair the healing process and indeed showed potential to accelerate wound closure [72].

Despite compelling in vitro evidence, the translation of bacteriocins from the laboratory to routine veterinary practice continues to face critical obstacles [70]. A key limitation remains the paucity of preclinical safety and biosafety data, which constitute an indispensable prerequisite for initiating clinical trials [69]. Combined with challenges related to delivery to the site of infection and a deficit of standardized farm-level studies, these factors effectively delay the entry of bacteriocins into clinical trials in target animal species [71]. For bioengineered peptides to become safe and useful veterinary therapeutics, interdisciplinary efforts will be required, integrating precise molecular design with advanced delivery technologies and rigorous safety profiling.

Beyond biological efficacy, successful implementation of bacteriocins in veterinary medicine will also depend on practical considerations. Species-specific dosing regimens remain insufficiently characterized for most candidate compounds, while regulatory requirements concerning withdrawal periods and residue monitoring have yet to be established for many bacteriocin-based therapeutics. Consequently, commercial adoption in livestock production will require not only demonstration of efficacy and safety, but also cost-effective manufacturing, scalable production processes, and clear regulatory pathways.

Although bacteriocins offer several advantages over conventional antibiotics, their practical implementation in veterinary medicine remains associated with important limitations. To facilitate a critical comparison between bacteriocins and conventional antibiotics in veterinary medicine, the principal advantages and limitations of both approaches are summarized in Table 2.

As shown in Table 2, bacteriocins offer several theoretical and experimentally supported advantages over conventional antibiotics, particularly regarding target specificity, biofilm activity, and reduced disruption of commensal microbiota. However, significant challenges related to pharmacokinetics, formulation, manufacturing, regulatory approval, and clinical validation currently limit their widespread implementation in veterinary medicine [45,64,65,73,74]. Consequently, bacteriocins should currently be regarded as promising complementary or alternative antimicrobial agents rather than direct replacements for conventional antibiotics. However, most of these advantages have been demonstrated under experimental conditions, whereas evidence from large-scale veterinary clinical studies remains limited.

### 3.4. Resistance to Bacteriocins: Mechanisms and Mitigation Strategies

Although bacteriocins are frequently regarded as promising alternatives to conventional antibiotics owing to their distinct mechanisms of action and relatively low incidence of cross-resistance, resistance development should not be considered negligible. As highlighted by [45], the emergence of bacteriocin-resistant bacteria remains possible and may ultimately limit the therapeutic utility of specific compounds if not adequately addressed. Therefore, the assumption that bacteriocins are inherently protected from resistance evolution requires careful consideration [45].

Resistance to bacteriocins may be either innate or acquired and appears to arise through multiple independent mechanisms [75]. Among the most frequently reported adaptations are alterations of the bacterial cell envelope that reduce bacteriocin binding, membrane insertion, or access to intracellular targets [75]. Additional mechanisms reported in the literature include active bacteriocin export through efflux systems [75] as well as enzymatic degradation of antimicrobial peptides [75]. Importantly, these adaptations may emerge through different evolutionary pathways even within closely related bacterial strains, highlighting the complexity of bacteriocin resistance [75].

Bacteriocin-producing bacteria have evolved dedicated immunity systems that protect them from the activity of their own antimicrobial products [75]. Genes encoding these immunity mechanisms are commonly located within bacteriocin biosynthetic gene clusters [75] and provide highly specific protection against cognate bacteriocins [75]. Although these systems primarily function as self-protection mechanisms [75], they demonstrate that bacterial populations possess the genetic capacity to develop effective defenses against bacteriocin-mediated killing [75].

Experimental evidence further supports the possibility of resistance evolution under selective pressure. The authors of [73] observed the emergence of resistant colonies following exposure to individual bacteriocins [73] and identified mutations affecting genes involved in bacteriocin uptake and transport, including *btuB, tolA,* and *tolC* [73]. These findings indicate that alterations in receptor and transport systems may substantially reduce susceptibility and should be considered when designing bacteriocin-based therapies [73].

Nevertheless, current evidence suggests that resistance development may be mitigated through rational therapeutic design [73]. In the same study, bacteriocin cocktails targeting distinct cellular entry pathways prevented the emergence of resistant mutants [73], whereas resistance readily developed during treatment with individual bacteriocins [73]. These observations indicate that combination strategies may represent a promising approach to resistance management [73]. However, the long-term stability of such effects and their relevance under clinical and field conditions remain insufficiently understood and require further investigation [73].

## 4. Current Veterinary Research Hotspots

### 4.1. Bacteriocins Against Biofilms

Biofilms remain one of the greatest challenges in veterinary treatment. Notably, bacteriocins show efficacy in eliminating biofilm-producing bacteria and can be seen as a remedy for infections caused by antibiotic-resistant strains [60,76,77,78,79,80,81]. Current research proves the effectiveness of bacteriocins produced by *Bacillus subtilis* at a concentration of 250 μL/mL against biofilms [76]; high sensitivity amongst *Staphylococcus* spp. and a lethal effect on certain *Streptococcus* spp. strains have been reported [76]. Efficacy against biofilms was observed even in the assessment of crude LAB bacteriocins which visibly limit the biofilm formation of *S. aureus* [81]. Researchers found that the addition of crude bacteriocin results in a lack of adherent cells in studied *S. aureus* [81]. In addition, enterocins produced by equine-derived *Enterococcus asini* and *Enterococcus saccharolyticus* have lately been proven to exhibit properties against *S. aureus* [77]. The mentioned bacteriocins compromise the growth of MRSA strains, therefore inhibiting their biofilms [77]. However, not only lesser-known bacteriocins are being researched for their biofilm-stopping abilities. Nisin has been looked into more thoroughly as well, showing the most encouraging inhibitory potential in MRSA growth and biofilm production [77]. Another bacteriocin derived from *L. lactis* which is effective in elimination of MRSA biofilm was described as L. l MK2 [77]. A class of novel bacteriocins in the context of biofilm inhibition are postbiotics produced by *Lactobacillus rhamnosus* KC3 isolated from camels’ milk [79]. Postbiotics not only exert general antibacterial activity against MRSA and CR-APEC (carbapenem-resistant avian pathogenic *E. coli*), but also affect biofilm production [79]. At subinhibitory concentrations, biofilm formation can be suppressed by more than 25% [79].

The well-known nisin has also been used in developing treatments for canine periodontal disease in in vivo studies [80]. By incorporating the bacteriocin within a guar gum biogel, the structure gains antimicrobial abilities, thus preventing bacterial dental attachment and biofilm formation [80]. Antimicrobial activity is maintained even in the presence of canine saliva or after two years of storage. Moreover, nisin–biogels exhibit great potential in controlling and eliminating mature biofilms while being safe for eukaryotic cells [80].

Novel bacteriocins and bacteriocins with properties enhanced by antibiotics exhibit capabilities to stop biofilm formation as well. In eradication of MRSA biofilm, hybrid bacteriocins ripcin C [82] and ripcin C_P23A_ [83] were effective. In the case of biofilm produced by MRSP strains, the highest efficacy was shown by the synergy of two bacteriocins and an antibiotic (the MP1-PenG-EnEJ97s combination: micrococcin P1 (MP1) and enterocin (EntjEJ97s)) [84]. Nevertheless, the majority of antibiofilm studies have been conducted in vitro, and the extent to which these findings translate to naturally occurring infections in animals remains insufficiently investigated.

### 4.2. Bovine Mastitis as a Translational Model

Bovine mastitis is an inflammatory disease of the mammary gland which poses economic expenses and, more importantly, a significant threat to public health and dairy production [60]. Mastitis can be caused by bacteria, such as *Staphylococcus* spp., *Streptococcus* spp., *Enterococcus* spp. and Gram-negative bacteria (e.g., *E. coli, Klebsiella* spp., *Enterobacter* spp.) [60,78]. The mentioned microorganisms are hazardous not only to livestock or other animals but also to humans and the environment according to the One Health concept [78]. The greatest attention should be given to bacterial strains which are described as antibiotic-resistant, especially MRSA [60] and MDR *E. coli* strains [78]. The foregoing approach in treatment assumed extensive use of antibiotics, which ultimately has led to an increase in resistance in bacteria as well as antibiotic residue accumulation in milk. As of this moment, the main focus of research regarding the disease is to conduct a steady search for alternatives to antibiotics effective against resistant strains [60]. Coming to the critical point, bovine mastitis treatment could be achieved by performing in vitro and in vivo tests concerning bacterial isolates’ resistance, efficiency of proposed bacteriocin treatment and their effects on target eukaryotic organisms.

Performing a comprehensive genomic analysis enables information on the basis of AMR and virulence potential of, e.g., MDR *E. coli*, to be obtained [78]. In total, there were 59 antimicrobial resistance genes (ARGs) found to play a significant role in AMR in *E. coli* mastitis isolates MBBL4 and MBBL5 [78]. Conservative genes encode resistance against aminoglycosides (e.g., *TolC, acrD, baeR*), beta-lactams (e.g., *acrB, CRP, evgA*), carbapenems (e.g., *marA, soxS*), cephalosporins (e.g., *acrA*), fluoroquinolones (e.g., *rsmA, emrA, emrR*), tetracyclines (e.g., *mdfA, emrY, emrK*) and vancomycin (e.g., *vanG*) [78]. Other detected genomic features contributing to the resistance were mobile genetic elements (MGEs, e.g., plasmids, prophages) [78] and virulence factor genes (VFGs, encoding, e.g., flagella, fimbriae, pili) [78]. By identifying resistance determinants, virulence factors, and mobile genetic elements, whole-genome sequencing may facilitate the selection of appropriate bacteriocins and help predict potential therapeutic limitations associated with specific MDR strains [78].

In the case of both *S. aureus* and *E. coli* infections, several immunological pathways, including HIF-1, IL-17, MAPK, and TNF signaling, were shared [85]. Comparative analyses of bovine mastitis models further identified transposable element-associated responses and suggested that selected transposable elements may serve as potential molecular markers of *S. aureus*-induced mastitis [85]. Such findings may contribute to a better understanding of host–pathogen interactions and support the future development of targeted antimicrobial approaches, including bacteriocin-based interventions [85]. As mentioned, multiple in vitro studies have been conducted in order to establish the use of bacteriocins as a method of treating mastitis. Bacteria isolated from bovine mastitis infections with the ability to form biofilms were susceptible to bacillocins [76,81], enterocins and bacteriocins produced by LAB bacteria, including nisin [77] and crude LAB bacteriocins [81]. In other in vitro tests, nisin’s wide spectrum of effectiveness has been proven against several Gram-positive bacteria causing mastitis (*S. aureus, B. cereus, E. faecalis, S. agalactiae, S. dysgalactiae, S. equi* subsp. *zooepidemicus*) with MICs of 2–16 μg/mL [86]. At the same time, lysostaphin has shown specific activity against *S. aureus* (MIC of 1 μg/mL) [86]. Similarly, recent in vitro assessments have confirmed that nisin and specific postbiotic substances (such as PS/EMo and PS/Eas) exhibit high antimicrobial activity against methicillin-resistant (MR) and methicillin-susceptible (MS) non-aureus staphylococci (NAS) and *Mammaliicoccus* spp. isolated directly from subclinical mastitis cases [87]. Mastitis isolates were also treated in vitro with the MP1-NisA-Aur combination. Several Gram-positive bacteria associated with bovine mastitis were susceptible to its activity, including *S. aureus, S. dysgalactiae,* and *S. uberis*, amongst others. The study found that the addition of AuresinePlus is fundamental in decreasing the MIC value of *S. aureus* independently of the duration of treatment [60]. MP1-NisA-Aur was also studied in vivo using two models: a murine skin infection model (paired with a bioluminescent MRSA strain) and a murine mastitis model. During the first assay, the bioluminescence signal was reduced, thus proving the effectiveness of bacteriocin combination in inhibition of *S. aureus* growth. In the mastitis in vivo model, no Gram-positive cells were detected in infected glands after receiving bacteriocin treatment. Furthermore, the glands showed a similar morphology to the uninfected control group [60].

The in vivo assessment of bacteriocins’ treatment efficacy in the murine model of mastitis has gained more interest lately. An interesting direction of research involves the use of non-aureus staphylococci (NAS) in inhibiting *S. aureus* in a superinfection model [88]. NAS strains contain bacteriocin gene clusters and are able to colonize the mammary glands [88]. *Staphylococcus capitis* 4231, *S. simulans* 3061 and *S. epidermidis* 1778 were found to be effective against *S. aureus* in in vitro tests [88]. The provided results qualified *S. simulans* 3061 (containing a lactococcin 972-like gene cluster) and *S. simulans* 1334 (containing lactococcin 972-like and lanthipeptide bacteriocin gene clusters) to further in vivo assessment [88]. In mouse mammary glands inoculated with a selected NAS strain and *S. aureus*, a reduction in *S. aureus* colonization was observed [88]. Models inoculated with the bacteriocin-producing bacteria showed a lower level of inflammation in the general cytokine profile when compared to mice inoculated with *S. aureus* [88]. However, limitations in this study persist: in the histological appraisal, the inflammation level remained similar in the case of both *S. simulans* isolates and the *S. aureus* superinfection model. In addition, the exact mechanism of inhibition in vivo cannot be assessed at the moment [88].

Another study which involved a mouse model of MRSA-induced mastitis assessed the efficiency of an engineered hybrid bacteriocin ripcin C_P23A_ [83]. Compared to streptomycin, the bacteriocin reduced the bacterial load as well as the inflammation of the infected mammary glands more efficiently [83]. Ripcin C_P23A_ exhibited anti-inflammatory properties at a smaller dose than streptomycin (5 mg/kg vs 10 mg/kg) with better results for myeloperoxidase activity [83]. The exact mechanism of inhibition of the inflammatory reactions by the bacteriocin involved the inhibition of the transcription of pro-inflammatory cytokines: TNF-α, IL-6 and IL-1β [83].

Collectively, the available evidence indicates that most bacteriocin-based mastitis studies remain at the in vitro or murine proof-of-concept stage. Although these models provide valuable information regarding antimicrobial efficacy, biofilm inhibition, and host responses, relatively few studies have been conducted directly in dairy cattle. Consequently, the translation of these findings into routine veterinary practice requires further validation in target species under field conditions.

Conducted research helps in understanding the bacterial pathogenesis and genetics behind bovine mastitis. Assessment of MRSA strains’ susceptibility to bacteriocins, in both in vitro and in vivo models, directs focus to novel pathways of treatment, crucial during the AMR challenge in veterinary medicine.

### 4.3. Engineered and Next-Generation Bacteriocins

Bacteriocins exhibit promising potential in the search for alternatives to antibiotics, thanks to not only their still-studied antibacterial and antibiofilm properties, but also their vulnerability to modifications [89]. Belonging to RiPPs [89], they can be post-translationally modified to display a desired mode of action and be active in a wider spectrum of activity [89]. When antimicrobial activities of specific functional groups are known, these structures can be used in creating a new type of compound, described under the names of engineered or next-generation bacteriocins [89].

Examples of newly synthesized macrocyclic lanthipeptides are thanacin and ripcin [82]. These novel bacteriocins are methyl-lanthionine analogs and can be produced using thanatin and rip-thanatin as templates. However, antimicrobial properties of these newly obtained bacteriocins were not satisfactory at first: they only inhibited growth of *Micrococcus flavus* [82]. To enhance their activity, ripcin was fused with the C-terminal end of nisin. Fusion resulted in the formation of a second-generation peptide, ripcin B-G, which exhibited stronger antibacterial properties against Gram-positive pathogens than sole nisin or ripcin [82]. Most importantly, ripcin B-G showed inhibitory activity against *S. aureus*, including a MRSA strain, and remained insensitive to the nisin resistance protein (a peptidase reducing activity of nisin) [82]. Moreover, newly generated lanthipeptides were active against Gram-negative bacteria; ripcin C was characterized by its inhibition of both Gram-positive and Gram-negative pathogens, establishing it as a bacteriostatic compound [82]. Continuing research on hybrid ripcin C, researchers designed further peptide mutations via mutagenesis on the C-terminal region, developing new mutants with anti-*S.aureus* activity: ripcin C_P23A_, ripcin C_I23A_, ripcin C_V23A_ and ripcin C_G23A_ [83]. Thanks to a single-amino-acid mutation, new peptides’ properties underwent significant changes. Among the engineered variants, ripcin C_P23A_ exhibited the highest antimicrobial activity against *S. aureus*, with MIC values that were 2- to 8-fold lower than those of the parental peptide ripcin C [83]. Ripcin C_P23A_ was even effective in eradicating MRSA biofilm at a much better efficiency rate than ripcin C (elimination of 75% of biofilm at a concentration of 8 mg/L, compared to the same percent elimination at concentration of 64 mg/L by ripcin C). Furthermore, the mutation also changed the nature of compounds: while ripcin C exhibited bacteriostatic characteristics, ripcin C_P23A_ showed complete eradication of MRSA strains at concentrations of 8 and 16 mg/L within 8 h [83]. Knowing that both compounds target lipid II, it can be argued that ripcin C_P23A_ may have the ability to disrupt the bacterial membrane, therefore establishing its bactericidal properties [83]. This assumption was later confirmed by a fluorescence microscopy assay, proving the effectiveness of single-amino-acid mutations in changing bacteriocins’ efficiency in killing bacterial cells. This correlates closely with recent biophysical models demonstrating how next-generation bacteriocins manipulate specific target cell envelope receptors and lipid matrices to orchestrate membrane disruption and subvert bacterial defenses [5]. As mentioned before, the effect of ripcin C_P23A_ on MRSA strains was also studied using a mouse mastitis model with satisfactory results [83]. The compound exhibited favorable biosafety, as evidenced by the absence of hemolytic activity against rabbit erythrocytes and the lack of cytotoxic effects toward RAW 264.7 macrophages, as well as good plasma stability, supporting its potential as a candidate for the treatment of *S. aureus*, particularly MRSA, infections [83].

As a matter of fact, not only Gram-positive bacteria are looked into in the context of novel bacteriocins. A major threat persisting in veterinary medicine is the abundance of AMR bacteria, including MDR Gram-negative bacteria, occurring in poultry. Research aiming to assess bacteriocins as a solid alternative in treatment involves engineering bacteriocins by recombining their domains [90]. One study analyzed bacterial isolates from a poultry slaughterhouse (derived from chicken gastrointestinal tracts and water) which inhibited growth of *E. coli* and *Salmonella* spp. As a result, using in vitro cell-free protein synthesis, bacteriocins were produced in controlled conditions. Obtained colicins E1, E7 and M were used to create hybrid colicins with an extended antimicrobial spectrum against MDR *E. coli* and *Salmonella* spp. [90]. The broadest antimicrobial activity was exhibited by hybrids ColE1-M, ColM-E1 and ColM-E7 [90]. Next-generation bioengineered bacteriocins, with refined modes of action, narrowed pathogen specificity, and broader activity spectra, represent a promising direction for AMR-resistant infection management.

### 4.4. Bacteriocins as Adjunct Therapies

Even though antibiotic therapies are becoming ineffective due to the rise of emergence in resistant pathogens, bacteriocins are not solely seen as their replacement. New strategies also rely on bacteriocin–antibiotic synergies to enhance the properties of both compounds in addition to gaining control over the AMR problem. Such combinations enlarge bacteriocins’ spectrum of activity, enable the use of lower concentrations of compounds and limit the increasing resistance amongst pathogenic bacteria [91]. Expanding on this multi-targeted approach, recent advancements demonstrate that multiplexed ‘cocktails’ combining different structural classes of bacteriocins (such as linear and circular peptides) can be rapidly produced to target specific pathogens, effectively eradicating multidrug-resistant strains while completely preventing the emergence of resistance [73].

Recent research suggests the use of synergy between the thiopeptide bacteriocin, micrococcin P1 (MP1), and rifampicin as a remedy for MRSA infections [91]. Using a murine skin infection model, the antimicrobial properties of the MP1–rifampcin mixture were revealed [91]. The synergistic combination was incorporated into a cream formula which was then applied to mice infected with bioluminescent *S. aureus* Xen31 [91]. In effect, the luminescent signal in treated mice declined after 24 h and did not reoccur during the span of 10 days [91]. Results indicate the efficacy of eradicating MRSA together with a long-lasting effect of MP1–rifampcin without developing resistance [91], in contrast to sole rifampicin and fucidin treatments in which the rise of resistance occurred [91].

*Staphylococcus pseudintermedius* poses another critical concern in veterinary medicine because of its methicillin resistance and biofilm-formation abilities [84]. An assay of antibiotic resistance occurring in *S. pseudintermedius* isolates from canine skin and soft tissue infections (SSTIs) was performed, with the results of the LMGT 4219 strain being chosen for further examination [84]. LMGT 4219 showed the highest level of resistance by not being susceptible to several groups of antibiotics (tetracycline, streptomycin, trimethoprim, ciprofloxacin, kanamycin, gentamicin, cloxacillin, clindamycin, erythromycin, ceftraxone and penicillin G (PenG)) [84]. Selected thiopeptides (micrococcin P1 (MP1) and enterocin (EntjEJ97s)) were analyzed to discover synergy with various antibiotics [84]. The results helped to translate the potential of MP1, PenG and EntEJ97s to the next phase of research. The combined compounds showed far more satisfactory antimicrobial activity against MRSP when compared to their independent use or commonly used fucidic acid. The three-component mixture was also highly effective in inhibiting MRSP biofilms [84]. Further tests involved in vivo assays in a murine skin wound infection model infected with methicillin-resistant *S. pseudintermedius.* The assay examined the efficacy of a cream containing the three studied compounds which was applied to mice [84]. After four treatments, the antimicrobial effect on MRSP was confirmed, with a significant decrease in bacterial cells in mice and results comparable to the well-known fucidin cream [84]. A similar study was performed to assess the efficiency of another bacteriocin–antibiotic mixture, GarKS-MP1-PenG (garvicin KS-micrococcin P1-penicillin G), against MRSA strains [92]. This combination was effective in elimination of MRSA and other Gram-positive pathogens (coagulase-negative staphylococci and *E. faecalis*), proving its broad spectrum of activity in skin infection treatment [92].

As a novel form of therapy against vancomycin-resistant strains of enterococci (VRE), treatment with the combination of antibiotics and bacteriocins was tested [93]. The study evaluated the high possibility of increasing the vulnerability of VRE strains by combining bacteriocins (pediocins from *P. acidilactici* and *P. pentosaceus* or enterocin produced by *E. faecium*) with antibiotics [93]. Combining compounds enhanced the antimicrobial effect on resistant strains: the inhibition of bacterial growth was observed at the level of 90% at the highest concentrations [93]. A long-term application of bacteriocins was assessed as well, in which bacteriocins inhibited VRE strains even after 30 days of exposure invariably [93]. These results indicate the lack of innate resistance mechanisms against bacteriocins in the studied strains [93]. The research suggests a possible synergistic effect which could be applied in treatments needing compounds with a specific spectrum of activity [93].

The research covering synergy between bacteriocins and antibiotics is not only limited to Gram-positive bacteria. Microcin C (McC), microcin J25 (MccJ25), microcin B17 (MccB17) and microcin E492 (MccE492) were proven to exert inhibition abilities against Enterobacteriaceae MDR and non-MDR strains (*E. coli*, *K. pneumoniae*, *S. enterica)* [94]. Their variability changed throughout the different strains from bacteriostatic to bactericidal, with the most promising results for McC and MccJ25 [94]. MccJ25 exhibited enhanced activity when combined with antibiotics; the most efficient combination involved synergy of MccJ25 + chloramphenicol and Mccj25 + colistin [94]. Beyond their direct antimicrobial effects, recent studies suggest that certain bacteriocins may also exert immunomodulatory activity, further supporting their potential as multifunctional therapeutic agents [95].

A new approach against resistant bacteria suggests another application of bacteriocins—in novel nanoconjugates. Incorporating bacteriocins within nanoparticles may help overcome limitations such as proteolytic degradation and reduced antimicrobial efficacy [96].

Among nanoparticle-based delivery systems, Solid Lipid Nanoparticles (SLNs) have attracted considerable attention due to their high stability and low toxicity. SLNs can encapsulate nisin, enhancing its delivery and biological activity [96].

From an antimicrobial perspective, SLN-encapsulated nisin demonstrated enhanced antimicrobial and antibiofilm activity against *Treponema denticola*, whereas AgNP–nisin conjugates showed improved activity against MDR *A. baumannii* and exhibited synergistic effects compared with the individual components [97].

Beyond antimicrobial applications, bacteriocin-based nanoconjugates have also shown anticancer potential. In vitro studies demonstrated anticancer activity of SLN–nisin formulations against HSC-3 oral cancer cells [96], while nanoparticle conjugation enhanced the antitumor activity of bacteriocins in MC3T3-E1 osteoblastic cells [98].

Bacteriocins exhibit a high therapeutic potential, especially in treatment of MRSA and MRSP infections. Combining them with antibiotics results in better efficacy of therapies and conclusive biofilm eradication along with reliability of the infections not emerging iterum. Nanoconjugates, on the other hand, provide a solution to bacteriocins’ limitations, giving hope to the future use of bacteriocins in practice. Although metagenomic approaches have considerably expanded the discovery pipeline for novel bacteriocins, the biological activity, safety, and practical applicability of many predicted molecules remain to be experimentally verified. In this context, the utilization of cell-free expression (CFE) systems combined with engineered DNA devices has emerged as a powerful platform to rapidly synthesize, optimize, and screen customized bacteriocin combinations against clinical pathogens, bridging the gap between genomic prediction and in vivo validation [73].

## 5. Translational Challenges of Bacteriocins in Veterinary Context

Despite the substantial body of experimental evidence demonstrating the efficacy of bacteriocins against veterinary pathogens, including MDR strains, their actual translation into clinical practice has remained strikingly limited.

### 5.1. Stability and Pharmacokinetic Limitations

Unlike conventional antibiotics, whose pharmacokinetic profiles are well established, bacteriocins—being peptidic in nature—are subject to a number of fundamental constraints in terms of bioavailability, stability and susceptibility to enzymatic proteolysis under physiological conditions [69,99]. A comparative assessment of four bacteriocins in a dynamic in vitro digestion model has shown that pediocin PA-1, bactofencin A and nisin all undergo considerable degradation during gastrointestinal transit, whereas microcin J25 by virtue of its distinctive lasso topology retains a measure of activity throughout this passage [100]. Following parenteral administration, bacteriocins encounter an altogether different array of obstacles, namely the proteases involved in hemostasis and fibrinolysis, which give rise to short plasma half-lives and an unpredictable biodistribution profile [100]. Class I lantibiotics, conferred a degree of protection by their rigid intramolecular thioether bridges of lanthionine and methyllanthionine, are partially shielded from proteolytic cleavage and exhibit greater thermal stability than their unmodified counterparts [101]. In veterinary practice, this translates into the fact that, beyond topical applications (such as intramammary, dermatological, or those confined to the gastrointestinal lumen), the systemic use of bacteriocins remains limited and requires advanced peptide-protection strategies. Novel strategies for future deployment include encapsulation, D-amino acid substitution or fusion to carrier proteins.

### 5.2. Regulatory Pathways for Veterinary Translation

A second set of barriers is regulatory in character and arises from what may be described as the dual identity of bacteriocins. On the one hand, nisin has held Generally Recognized As Safe (GRAS) status for several decades; moreover, it is approved by the FDA, the EFSA and regulatory authorities in upwards of eighty countries as a food preservative (E234) [100]. On the other hand, its use as a veterinary medicinal product demands a markedly more exacting regulatory pathway. Within the European Union, it is regulated in accordance with Regulation (EU) 2019/6, and in the United States through the New Animal Drug Application (NADA) procedure overseen by the FDA Center for Veterinary Medicine. This duality of status—established safety as a food additive on the one hand, and the requirement for full clinical and pharmaceutical assessment as a veterinary medicinal product on the other—is itself a source of considerable translational delay. The case of the nisin-based intramammary preparations developed by ImmuCell Corporation is instructive in this regard: Wipe Out^®^ (a teat disinfectant) duly secured FDA approval, whereas Mast Out^®^ (an intramammary product intended for the treatment of subclinical mastitis) has remained within the NADA pipeline for well over a decade [100]. A further obstacle stems from the absence of any harmonized regulatory pathway for RiPP-class peptides in veterinary applications. Bacteriocins still may variously be classified as medicinal products, feed additives or biological agents, with each category carrying its own distinct regulatory requirements [70].

### 5.3. Clinical Evidence Gap

The third barrier concerns a striking gap in clinical evidence. The overwhelming majority of available data on bacteriocin activity in the veterinary context derive from in vitro studies and animal models, most often murine, far less frequently from the target species themselves, such as dairy cattle or dogs [69]. Randomized controlled clinical trials in target animal species remain few and far between. In the case of nisin for bovine mastitis, the field continues to rely, in essence, upon a single, albeit methodologically robust study [55], whilst contemporary evidence for nisin derivatives and novel bacteriocins is for the most part confined to murine models. The lack of standardized assessment protocols, encompassing harmonized methods for in vivo MIC determination, pharmacokinetic models for target species, and phase III trials, hampers cross-study comparison and serves only to delay regulatory decision-making [71,102]. Bridging this gap will require coordinated, farm-level studies that assess not only clinical efficacy, but also impacts on animal microbiomes, residues in animal-derived products and economic feasibility. Without such data, bacteriocins are likely to remain promising laboratory molecules rather than validated therapeutic tools in veterinary practice.

## 6. Future Directions

While the potential of bacteriocins as antimicrobial agents is well documented, the recent veterinary-focused studies summarized in Table 3 highlight a critical transition point in the field. Although efficacy against key pathogens has been demonstrated across diverse models, the translational hurdles and limitations noted in Table 3—such as proteolytic instability, the need for formulation optimization, and a lack of farm-level clinical trials—indicate that future progress will require coordinated efforts in three complementary areas: disruption of biofilm tolerance [26], exploration of the animal microbiota [103,104,105,106,107] and precision therapy [66,108] as a source of novel antimicrobial molecules.

### 6.1. Precision Antimicrobials

The development of stimuli-responsive nanocarriers offers the prospect of controlled bacteriocin release within specified tissues, or in response to particular molecular cues—be it the lowered pH that accompanies infection, the presence of bacterial enzymes, or markers of inflammation. In the veterinary context, such an approach raises the possibility of therapies tailored to a specific pathogen and infection site, whilst reducing systemic dosing and limiting collateral effects upon the commensal microbiota of the host [66]. 

### 6.2. Biofilm-Targeted Therapies

A second line of development centers on overcoming biofilm tolerance—a key driver of therapeutic failure in bovine mastitis, canine otitis externa, and implant-associated infections. Strategies under investigation include the coating of veterinary implant surfaces with bacteriocin-eluting layers, as well as combinations of bacteriocins with agents that destabilize the EPS matrix, thereby permitting synergistic penetration and disruption of the biofilm architecture [26].

### 6.3. Microbiota-Based Strategies

Among the most rapidly advancing directions is the exploration of the animal microbiota as a rich source of novel bacteriocins, naturally tailored in their spectrum of activity. A systematic screening of 441 non-aureus staphylococci (NAS) isolates from bovine milk identified 40 strains, spanning nine species, including *S. simulans, S. chromogenes, and S. epidermidis*, capable of inhibiting *S. aureus*, with 23 of these also active against MRSA [103]. A complementary investigation of producers of bacteriocin-like inhibitory substances (BLISs), peptides exhibiting antibacterial activity and protease sensitivity but awaiting full biochemical characterization, conducted on 890 staphylococcal isolates of varied origin, further confirms that the animal microbiota represents an underexploited reservoir of antimicrobial peptides [104]. A particularly promising example is caledonicin, a 64-amino-acid circular bacteriocin recently identified in *Staphylococcus caledonicus* derived from the canine microbiota, and exhibiting broad-spectrum activity against MRSA and methicillin-resistant *S. pseudintermedius* (MRSP) [105]. A further emerging avenue concerns the modulation of the rumen microbiota in ruminants, integrating therapeutic and environmental objectives, including a reduction in methane emissions, of which enteric fermentation in the rumen represents a leading agricultural source [106]. Such an approach broadens the One Health framework to encompass an environmental dimension, integrating animal health, food safety, and sustainable agricultural production [71].

## 7. Conclusions

Bacteriocins represent a class of molecules with exceptionally well-documented mechanistic diversity, a broad spectrum of clinical applications, and promising preclinical evidence in veterinary medicine. Their molecular distinctiveness from conventional antibiotics, ability to overcome biofilm-associated tolerance, and low frequency of cross-resistance make them attractive candidates in an era of escalating antimicrobial resistance and increasingly restrictive regulatory requirements.

Despite these advantages, their implementation in veterinary practice remains limited by pharmacokinetic, regulatory, and evidentiary challenges. Addressing these barriers will require coordinated efforts spanning biotechnology, clinical pharmacology, and regulatory science.

Future progress depends not only on continued mechanistic and bioengineering research, but also on well-designed clinical trials in target animal species, harmonized approval pathways for RiPP-class peptides, and further exploration of the animal microbiota as a source of novel antimicrobial compounds with species-specific activity profiles.

Within the One Health concept, bacteriocins could become an important tool for preserving the effectiveness of antimicrobials critical to human medicine. Achieving this goal will depend on successful integration of scientific advances into veterinary practice and regulatory frameworks.

## Figures and Tables

**Table 1:** Selected antimicrobial classes of critical importance in veterinary medicine and clinical indications for which no equivalent alternatives are available. Adapted from the WOAH *List of Antimicrobial Agents of Veterinary Importance* (2025) [13].

Class of Antibiotics (WOAH Category)	Indication Without Alternatives	Target Species	Reference
Fluoroquinolones (HPCIA)	Colibacteriosis, mycoplasmosis, septicemia	Poultry, livestock, swine	[13]
Cephalosporins III/IV gen. (HPCIA)	Metritis, septicemia; mastitis (applied topically)	Livestock, swine, canines, felines	[13]
Colistin (HPCIA)	Colibacteriosis, intestinal infections (*E. coli*)	Poultry, livestock, swine	[13]
Fosfomycin (HPCIA)	Intestinal and systemic infections	Livestock, swine, poultry, fish	[13]
Macrolides (VCIA)	*Mycoplasma* spp. infections	Poultry, livestock, swine	[13]
Pleuromutilins (VCIA)	Swine dysentery (*Brachyspira hyodysenteriae*)	Swine, poultry	[13]

**Table 2:** Critical comparison of conventional antibiotics and bacteriocins in veterinary medicine.

Feature	Conventional Antibiotics	Bacteriocins	Supporting Reference(s)
Spectrum of activity	Often broad-spectrum	Frequently narrow-spectrum and targeted	[45]
Resistance development	Common and extensively documented	Lower frequency reported, although adaptive resistance mechanisms and resistance evolution have been described	[45,73]
Activity against biofilms	Often substantially reduced in mature biofilms	Frequently retain activity against biofilm-associated cells	[25,45]
Impact on microbiota	May induce dysbiosis and collateral microbiota disruption	Generally lower collateral impact due to narrower spectrum	[45,64,65]
Pharmacokinetics	Well characterized for most veterinary drugs	Often limited by proteolytic degradation, short half-life, and low bioavailability	[74]
Formulation requirements	Numerous established formulations available	Frequently require encapsulation, peptide engineering, or advanced delivery systems	[74]
Manufacturing and production	Mature industrial production platforms available	Production, purification, and large-scale manufacturing remain challenging for many bacteriocins	[74]; current review synthesis
Regulatory approval	Extensive veterinary approval pathways and established guidelines	Limited regulatory experience and very few approved veterinary products	Current review synthesis
Withdrawal periods and residue monitoring	Well-established procedures and legal frameworks	Limited information available for many candidate bacteriocins	Current review synthesis
Clinical evidence in veterinary medicine	Extensive field and clinical experience	Predominantly in vitro, murine, and preclinical evidence, with relatively few target-species studies	Current review synthesis (Table 2)

**Table 3:** Recent veterinary-focused bacteriocin studies.

Bacteriocin	Source	Target Pathogen	Veterinary Context	Development Stage	Model	Administration Route	Key Result	Dosage/ Formulation	Limitation
NZ2114	[57]	MRSP	Treatment of canine superficial pyoderma	In vivo	Murine superficial pyoderma model	Intraperitoneal injection	Decrease in the number of skin bacteria, reduction in the skin damage area, biofilm inhibition	5 mg/kg	Need for further research to optimize preparation; anatomical differences (translational limitation)
MP1-NisA-Aur	[60]	MRSA	Treatment of bovine mastitis	In vivo	Murine skin infection model, murine mastitis model	Intramammary injection	Elimination of biofilm	10 mg/mL MP1, 1 mg/mL NisA, 1 mg/mL Aur	Observed relapse of infection; anatomical differences (translational limitation)
Bacillocin from *B. subtilis*	[76]	MRSA, *Streptococcus* spp.	Treatment of bovine mastitis	In vitro	Bacterial (agar well-diffusion assay)	Not applicable	High-efficacy biofilm elimination	250 µg/mL	Vulnerability to proteases in teat skin
Enterocin from *E. asini* EAs 1/11D27	[77]	MRSA	Treatment of livestock, wildlife MRSA infections	In vitro	Bacterial (agar spot test)	Not applicable	High efficacy in biofilm elimination on multiple MRSA strains	100–200 AU/mL	Susceptibility varies between MRSA strains
Enterocin from *E. saccharolyticus* Es3/11D27	[77]	MRSA	Treatment of livestock, wildlife MRSA infections	In vitro	Bacterial (agar spot test)	Not applicable	High efficacy in biofilm elimination on multiple MRSA strains	100–800 AU/mL	Susceptibility varies between MRSA strains
L. l MK 2 from *Lactococcus lactis*	[77]	MRSA	Treatment of livestock, wildlife MRSA infections	In vitro	Bacterial (agar spot test)	Not applicable	High efficacy in biofilm elimination on multiple MRSA strains	100–200 AU/mL	Susceptibility varies between MRSA strains
Nisin from *Lactococcus lactis*	[77,80]	MRSA, *Enterococcus* spp.	Treatment of livestock, wildlife MRSA infections, treatment of canine periodontal disease	Clinical trials	Canines	Biogel	Development of effective nisin–biogel medication	200 µg/mL	A suspected development of resistance in *Enterococcus* spp.
Caledonicin from *S. caledonicus*	[105]	MRSA, MRSP, *L. monocytogenes,* *C. difficile*	Treatment of canine infections	In vitro, in silico	Bacterial (agar spot test)	Not applicable	Use of effective bacteriocins derived from canine microbiota	Concentration not specified, 2 μL	Need for research expansion on numerous samples
NAS-derived bacteriocins, e.g., from *S. simulans*	[88]	*S. aureus*	Treatment of bovine mastitis	In vivo	Murine superinfection model	Intramammary injection	High efficacy in *S. aureus* inhibition	400 CFU of *S. simulans* against 100 CFU of *S. aureus*	High inflammation in histological appraisal; faintly familiar mechanism of inhibition; anatomical differences (translational limitation)
Bacteriocin *S. pseudintermedius* E18	[107]	MRSA	Potential treatment of MRSA infections	In silico analysis, in vitro	Bacterial	Not applicable	An extremely narrow spectrum of activity targeted at destroying pathogenic, methicillin-resistant *Staphylococcus aureus* (MRSA) strain	0.05 ± 0.02 µg/mL	Need for further research to characterize the bacteriocin
Ripcin C_P23A_	[83]	MRSA	Treatment of bovine mastitis	In vivo	Murine mastitis model	Intramammary injection	Bactericidal properties, more effective than previous bacteriocin mutants	20 mg/kg	Several steps of production
ColE1-M, ColM-E1 and ColM-E7	[90]	*E. coli,* *Salmonella* spp.	Treatment of poultry MDR infections	In vitro	Bacterial (agar spot test)	Not applicable	An extended spectrum of activity compared to native colicins	10 ng/µL in 25 µL reaction volumes	Inactivation by proteases; reduced stability under processing conditions; need for safety validation
MP1 + rifampicin	[91]	MRSA	Treatment of bovine mastitis	In vivo	Murine skin infection model	Topical (skin cream)	A long-lasting reduction with no resistance, eradicating biofilm	0.01 mg/mL of MP1, 0.15 mg/mL of rifampicin	Further research is needed (to understand the molecular mechanisms behind the synergistic effects); anatomical differences (translational limitation)
MP1 + PenG + EntEJ97s	[84]	MRSP	Treatment of MRSP infections	In vivo	Murine skin infection model	Topical (skin cream)	Inhibiting MRSP biofilms	5.0 mg/mL of PenG, 0.2 mg/mLof MP1, 1 mg/mL of EntEJ97S	Need for biofilm assays in vivo and investigation of mechanism of activity against biofilms; anatomical differences (translational limitation)
GarKS + MP1 + PenG	[92]	MRSA	Treatment of MRSA infections	In vivo	Murine skin infection model	Topical (gel)	High efficiency in eradicating bacteria from wounds	0.1 mg/mL MP1, 5 mg/mL garvicin KS, and 5 mg/mL penicillin G in 5% hydropropylcellulose	Need for optimization of the formulation; anatomical differences (translational limitation)
Pediocin + vancomycin, enterocin + vancomycin	[93]	VRE	Treatment of VRE canine infections	In vitro	Bacterial (antimicrobial susceptibility testing)	Not applicable	High specificity and a promising inhibitory effect against VRE	200 AU mL^−1^	Further research to assess the spectrum of activity
MccJ25 + chloramphenicol, Mccj25 + colistin	[94]	*E. coli, K. pneumoniae, S. enterica*	Treatment of MDR Enterobacteriaceae	In vitro	Bacterial	Not applicable	Antagonism and enhancing effect between microcin and antibiotics	0.02–42.5 μM	Poorly studied natural resistance emergence to microcins
Vancomycin + lentuscin	[108]	*S. aureus,* *S. epidermidis*	Treatment of *Staphylococcus spp.* infections and tumors	In vitro	Bacterial, A375 melanoma cell line	Not applicable	Cancerous cells are specifically targeted by the bacteriocin	5–200 μg mL^−1^	Further research to understand the mechanisms and for in vivo validation
Bacteriocins + nanoparticles (SLN–nisin, CeO_2_–bacteriocin nanohybrid, AgNPs–nisin)	[96,97,98]	*T. denticola* (SLN–nisin), *S. epidermidis* (CeO_2_NPs–bacteriocin), *A. baumanii* (AgNPs–nisin)	Treatment of periodontal disease (SLN–nisin) and bacterial infections involving biofilm	In vitro	Bacterial, cell lines: HSC-3 for SLN–nisin, MC3T3-E1 for CeO_2_NPs–bacteriocin	Not applicable	Biofilm inhibition, anticancer properties in cell lines	0.25 μg/mL^−1^ μg/mL (SLN–nisin), 31.25 µg/mL (CeO_2_NPs–bacteriocin), 125–52 µg/mL (AgNPs–nisin)	Need for in vivo assays

## Data Availability

No new data were created or analyzed in this study. Data sharing is not applicable to this article.
